# Case Report: Spinal cord abscess due to *Nocardia farcinica* presenting as longitudinally extensive transverse myelitis

**DOI:** 10.3389/fmed.2025.1613770

**Published:** 2025-06-05

**Authors:** Tayoot Chengsupanimit, Amit Mahajan, Shelli Farhadian, Francisco Machiavello Roman

**Affiliations:** ^1^Section of Infectious Diseases, Department of Medicine, Yale University School of Medicine, New Haven, CT, United States; ^2^Department of Radiology and Biomedical Imaging, Yale University School of Medicine, New Haven, CT, United States

**Keywords:** transverse myelitis, nocardiosis, spinal abscess, metagenomic next-generation sequencing, solid organ transplant

## Abstract

A middle-aged man, renal transplant recipient, was admitted with lower extremity paralysis, loss of sensation and urinary retention. The initial diagnostic workup revealed extensive inflammatory spinal changes on imaging, consistent with longitudinally extensive transverse myelitis. Cerebrospinal fluid testing demonstrated neutrophilic pleocytosis; routine tests for bacterial and viral pathogens were negative. The patient received high-dose steroids for presumed autoimmune myelitis, but his condition worsened. Repeat spinal imaging revealed an intramedullary spinal cord abscess and a loculated collection in the cauda equina. *Nocardia farcinica* was isolated from spinal biopsy tissue cultures and metagenomic sequencing of cerebrospinal fluid. He received treatment with trimethoprim-sulfamethoxazole and linezolid, with subsequent improvement of the radiological abnormalities. At outpatient follow-up two months after initiating antimicrobials, the patient endorsed improved upper extremity strength, though remained paraplegic. This case report highlights the protean manifestations of central nervous system nocardiosis and the benefits of using metagenomic sequencing to diagnose complex central nervous system infections.

## Introduction

Infections by *Nocardia* species predominantly affect immunocompromised subjects, in whom they present with variable clinical manifestations. A distinctive feature of the species is its neurotropism and propensity to cause central nervous system infections. Although predominantly presenting as brain abscesses, other forms of central nervous system involvement, such as meningitis and spinal abscesses, have also been described.

This article describes an unusual presentation of spinal nocardiosis as longitudinally extensive transverse myelitis in a solid organ transplant recipient and highlights the diagnostic challenges associated with this entity. To our knowledge, this is the first case of spinal nocardiosis presenting as longitudinally extensive transverse myelitis described in the medical literature, as opposed to the more common presentation of well-defined spinal abscesses.

Furthermore, we present evidence supporting the use of metagenomic next-generation sequencing early in the diagnostic workup of patients with complex neurologic infections. In this patient’s case, next-generation sequencing allowed for a microbiological diagnosis before obtaining results from an invasive tissue biopsy and culture.

## Case presentation

**Figure 1:**
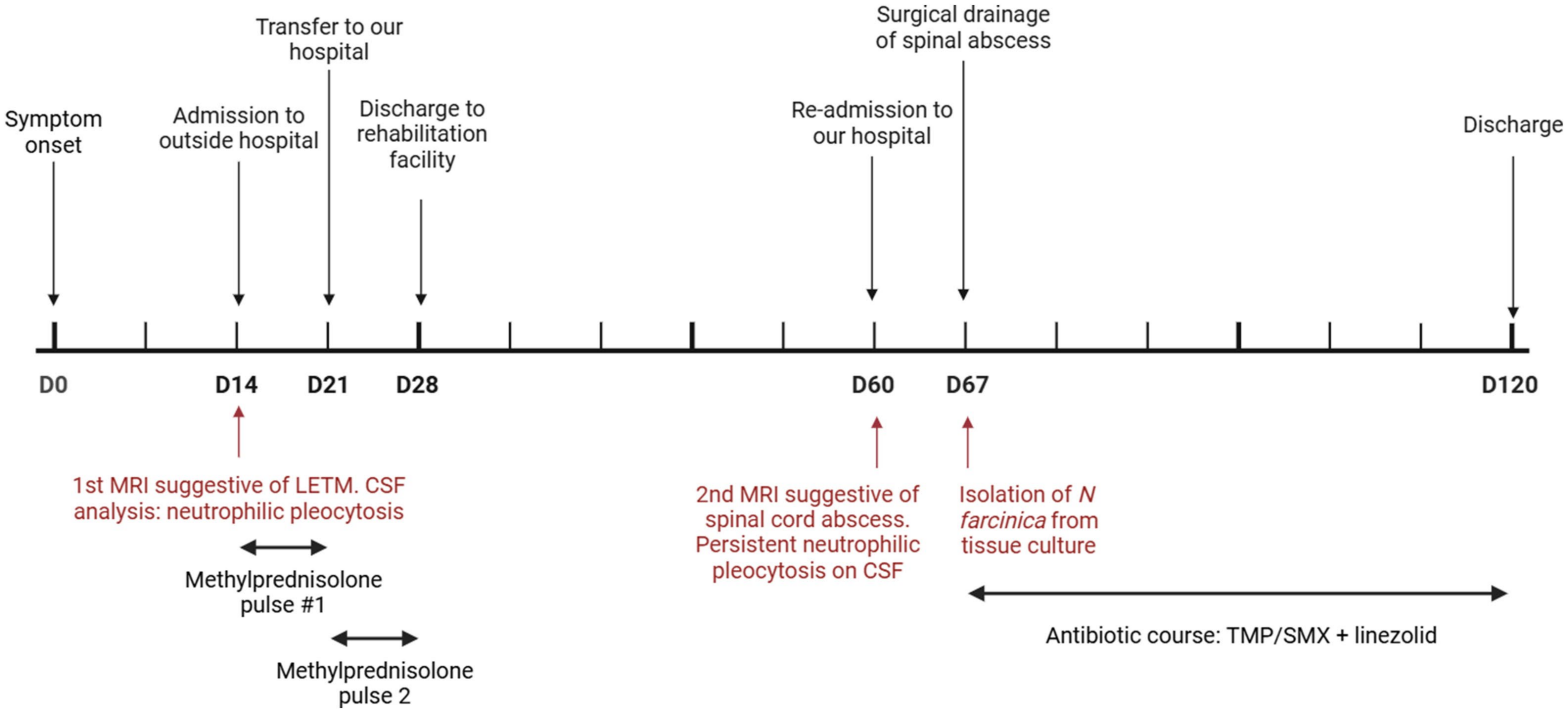
Clinical course timeline. LETM: longitudinally extensive transverse myelitis.

A 57-year-old man was admitted to an outside hospital with lower extremity paralysis, loss of sensation, and urinary retention (clinical course summarized in Figure 1). His symptoms developed over two weeks and acutely worsened two days before admission. The patient denied any fever, chills, or preceding spinal trauma. Notably, one month prior to this admission, he had been admitted on two separate occasions for pain and numbness of his right lower extremity and was found to have a large thrombus from the superficial femoral to the popliteal artery – the thrombosis was thought to be the cause of his symptoms, and, as he was not a candidate due to the extensive clot burden, he was treated with anticoagulation alone and discharged.

His medical history was relevant for hypertension, peripheral artery disease, and type 2 diabetes mellitus complicated by end-stage renal disease, for which he had undergone kidney transplantation ten years before admission. His post-transplant course was complicated by an episode of acute cellular rejection, treated with pulse doses of methylprednisolone and a prolonged prednisone taper three months before symptom onset. He took tacrolimus and prednisone for immunosuppression and was not on any antimicrobial prophylaxis at the time of admission. Other relevant medications included metformin, chlorthalidone and apixaban. He was born and raised in Connecticut, United States, and had recently retired from a twenty-year career in city maintenance. He had no pets but reported exposure to feral cats. He had not recently traveled and denied consumption of raw or unpasteurized products.

On presentation, his blood pressure was 110/60 mmHg, heart rate 86/min, respiratory rate 14/min and temperature 36.3C°. His exam was remarkable for symmetric lower extremity paresis (0/5 proximal and distal), diminished muscular tone, absent deep tendon reflexes, and an L2 sensory level. Cranial nerve function, upper extremity strength, coordination, and sensation were preserved.

**Figure 2:**
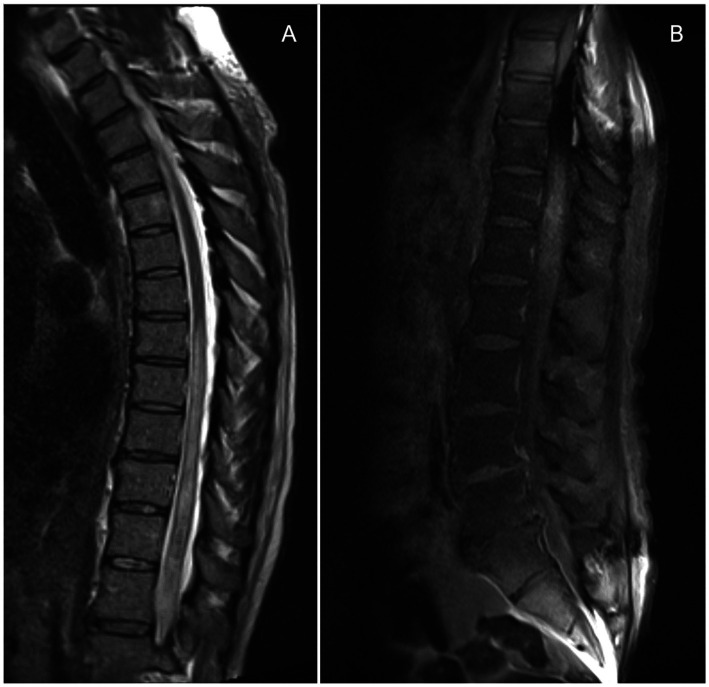
Sagittal T2 **(A)** and postcontrast sagittal **(B)** T1 weighted images on first admission showing abnormal T2 signal involving the thoracic spinal cord extending to the conus medullaris, with faint postcontrast enhancement consistent with acute myelitis.

Laboratory tests showed a normal white blood cell count (9.2×10^9^/L, 75% neutrophils, 15% lymphocytes), stable renal function (creatinine 1.29 mg/dL), and a therapeutic blood tacrolimus concentration (7.8 ng/mL); the rest of the complete blood count and metabolic panel were within normal limits. Spinal magnetic resonance imaging (MRI) identified diffuse T2 hyperintensity within the central cord with peripheral enhancement of the thoracic spinal cord and extending caudally into the conus, favored to represent an inflammatory process consistent with longitudinally extensive transverse myelitis (Figure 2). A cerebrospinal fluid (CSF) analysis showed 223 total nucleated cells/uL (97% granulocytes, 1% lymphocytes), glucose 66 mg/dL and protein 190 mg/dL; a Gram stain and culture were negative. The initial diagnostic workup for infectious etiologies (CSF HSV, VZV, EBV and CMV PCRs; West Nile Virus IgM and IgG, and cryptococcal antigen; blood Lyme, HTLV-1, and *T pallidum* serology) and autoimmune conditions (antinuclear antibodies, antineutrophil cytoplasmatic antibodies, rheumatoid factor, aquaporin-4 antibody) was negative.

He received a five-day course of methylprednisolone 1 g IV daily for suspected transverse myelitis without any symptomatic improvement, prompting his referral to our hospital for consideration of plasmapheresis. He received a second five-day course of methylprednisolone, with an interim minimal improvement of lower extremity sensation and proximal lower extremity strength; hence, plasmapheresis was not pursued. The subtle improvement was attributed to reduced spinal cord edema; he was expected to recover some motor strength with intense rehabilitation. He was discharged to a rehabilitation facility to continue physical therapy.

**Figure 3:**
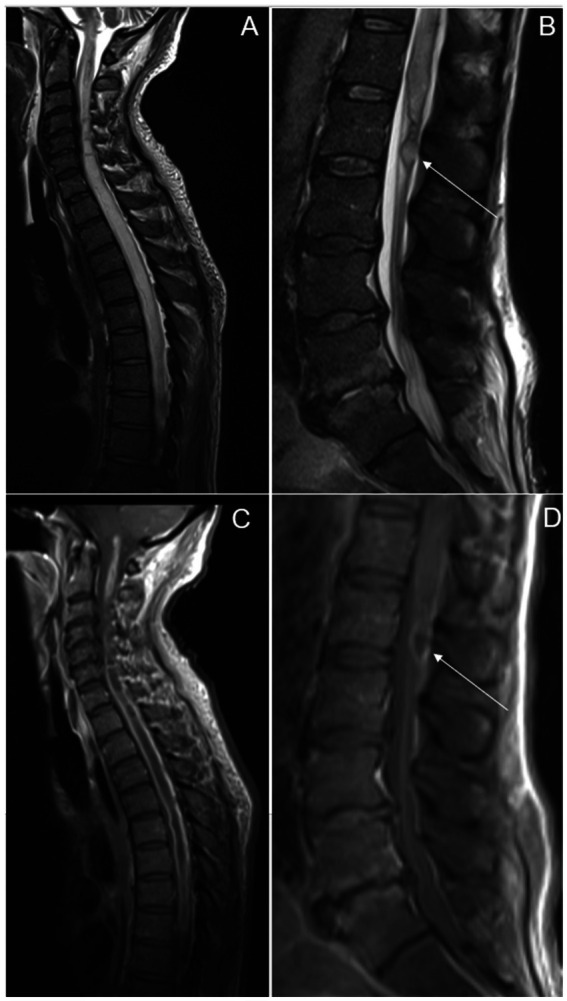
Sagittal T2 weighted images obtained 1 month later of **(A)** cervical and thoracic and **(B)** lumbar spine showing worsening T2 signal abnormality of the spinal cord, as well as a localized fluid collection in the region of the cauda equina (arrow). Post contrast images **(C,D)** reveal extensive enhancement of the cervical and thoracic spinal cord as well as rim enhancing collection within the cauda equina (arrow).

He was readmitted one month later with persistent lower extremity weakness and new right upper extremity paresis. A repeat spinal MRI demonstrated interval progression of spinal cord edema with expansion extending from the obex of the fourth ventricle to the conus medullaris, an intramedullary spinal cord abscess extending from the craniocervical junction to the lower thoracic region, and a 1.5 cm rim-enhancing loculated fluid collection in the cauda equina caudal to the conus medullaris (Figure 3). There was enhancement of the cauda equine nerve roots and other enhancing nodules within the subarachnoid space. These findings were interpreted to represent large spinal abscesses, prompting a shift in the patient’s care from treating a primary inflammatory process to an invasive CNS infection.

A repeat CSF analysis revealed persistent neutrophilic pleocytosis 20 total nucleated cells/uL (81% granulocytes, 8% lymphocytes), glucose 83 mg/dL and protein 1776 mg/dL, but the cultures were negative; a sample was sent out for metagenomic next-generation sequencing (mNGS, Mayo Clinic). While awaiting the results of the mNGS, surgical drainage of the loculated collection within the cauda equina was pursued. Intraoperatively, the entire spinal cord was encased in nodular yellow material, and when the collection was opened, thick, copious yellow purulence was encountered. This fluid was aspirated and consisted of dense polymorphous inflammatory infiltrates with numerous neutrophils, an abundance of macrophages and admixed lymphocytes, and branching Gram-positive rods on Gram stain. The mNGS was exclusively positive for *Nocardia farcinica*, which was also isolated a few days later from the abscess culture. The tissue sample was inoculated in blood and buffered charcoal-yeast extract agars under aerobic conditions at 37°C. Chalky white colonies were isolated within 48 h and identified as *Nocardia farcinica* through MALDI-TOF mass spectrometry. Broth microdilution susceptibility testing identified that the isolate was susceptible to trimethoprim-sulfamethoxazole, linezolid, moxifloxacin, amoxicillin-clavulanic acid and amikacin and displayed resistance against imipenem, ceftriaxone, doxycycline and clarithromycin.

The patient received linezolid 600 mg orally twice daily and trimethoprim-sulfamethoxazole 320/1600 mg orally every eight hours; the latter provoked hyperkalemia, and hence, the dose was reduced to 320/1600 mg twice daily. A spinal MRI obtained one month after starting treatment showed a decreased cord abscess, with a decreased rim-enhancing conus medullaris collection size and a marked decrease in cord expansion. The patient’s motor and sensory deficits persist, and he is expected to continue rehabilitation therapy in the outpatient setting. At outpatient follow-up two months after initiating antimicrobials, the patient had sustained improvement in upper extremity strength, though he remained paraplegic.

## Discussion

We present a challenging case of spinal infection by *Nocardia farcinica* in an immunocompromised host that initially presented radiologic features of longitudinally extensive transverse myelitis. Although it is difficult to ascertain, we hypothesize that earlier recognition of this patient’s presentation as the result of spinal nocardiosis, instead of a primary inflammatory process, would have led to quicker antibiotic initiation and more substantial neurologic recovery.

Longitudinally extensive transverse myelitis (LETM) is a rare yet severely disabling condition characterized by a lesion of the spinal cord that extends over three or more vertebral segments. In our patient, this manifested as paralysis of the bilateral lower extremities and his right upper extremity. Although it is most commonly associated with neuromyelitis optica, the differential diagnosis includes multisystem autoimmune inflammatory diseases such as systemic lupus erythematosus or sarcoidosis, neoplasia such as B-cell lymphoma or intramedullary spinal cord tumors such as ependymomas or spinal astrocytomas, and infection (1). Multiple organisms have been implicated as direct agents of spinal infection causing LETM, such as syphilis, neuroborreliosis, or HTLV-1; in other instances, LETM may be triggered as a post-infectious manifestation of systemic HIV, VZV, EBV, CMV, and dengue infections (2). Although spinal involvement in nocardiosis has been described before (3), to our knowledge this is the first report of longitudinally extensive involvement due to spinal nocardiosis.

*Nocardia* spp. is an environmentally ubiquitous, partially acid-fast, branching filamentous genus of Gram-positive bacteria found worldwide. Many species within the *Nocardia* genus are clinically relevant, including *N. farcinica*, *N. cyriacigeorgica* and *N. nova* complex (3). Its portal of entry is typically the respiratory tract, after which systemic dissemination may occur, most commonly among immunocompromised hosts. Rates of disseminated disease in solid organ transplant recipients have been reported between 20–71% (3). The *Nocardia* species display important neurotropism that usually manifests as brain abscesses. Previous case series have estimated that one-third of solid organ transplant recipients with nocardiosis have brain involvement (3). Spinal involvement is much rarer – one series found only a total of 26 cases of *Nocardia* spp. spinal abscess in the literature (4). In this series, 46% had spinal epidural abscess, 42% had intramedullary abscess, and 12% had both.

*Nocardia* spp. is of specific concern when encountering neurological findings in patients who have undergone solid organ transplantation (3). Immunity against *Nocardia* is primarily T-cell-mediated (5), making patients with impaired T cell immunity, such as those with solid organ and hematopoietic stem cell transplant populations, patients living with HIV, lymphoreticular neoplasias, or those treated with chronic corticosteroids, particularly susceptible (3). An episode of acute rejection within 6 months before diagnosis and use of high-dose corticosteroids were also independent risk factors that significantly increased the risk of nocardiosis (6, 7). Our patient was on chronic immunosuppression and had also been treated with pulse-dose steroids, including a taper for acute cellular rejection three months before the development of his initial paraplegia, which put him at high risk of *Nocardia* spp. infections. However, strategies for reducing the risk of nocardiosis during periods of intensified immunosuppression have not been established. Breakthrough *Nocardia* infections have been well-documented despite the use of trimethoprim-sulfamethoxazole prophylaxis (3). Hence, *Nocardia* spp. infections should always be considered in at-risk patients presenting with neurologic symptoms regardless of their antimicrobial prophylaxis.

As illustrated in this case, delays in diagnosing nocardiosis are common due to the nonspecific nature of the illness and challenges in obtaining invasive tissue samples for culture. Metagenomic next-generation sequencing of the CSF has the potential to quicken the time to diagnosis of CNS infections. In a recent study of seven years of experience at a large academic medical center, 48 out of 220 diagnoses of a CNS infection were made by mNGS alone (21.8%), and the specificity of mNGS testing for CNS infections was 99.6% (8). This study included one instance wherein *Nocardia nova* was only detected by mNGS and not by standard culture methods. Additional reports support the use of mNGS in diagnosing pulmonary, disseminated, and CNS nocardiosis based on the quicker turnaround time of mNGS compared to standard microbiological techniques, including less common *Nocardia* species (9–11). A study by Weng et al. reported that 9 out of 25 samples (36%) from 21 patients, including CSF, bronchioalveolar lavage and lung tissue, were positive by next-generation sequencing but not by culture, suggesting that mNGS may play a substantial role in cases where culture fails to make a diagnosis (12). Its use early in the course of a complex case should be strongly considered, since it may establish a diagnosis before standard microbiological methods. The major limitation of mNGS compared to standard culture is that antimicrobial susceptibility cannot be determined by mNGS alone, hence, empiric antimicrobial courses may be warranted when *Nocardia* spp. is not recovered from cultures.

Our patient’s presentation illustrates a case of spinal cord abscess associated with arachnoiditis due to *Nocardia farcinica*. A unique feature was the initial radiological presentation of this condition as LETM, which led to initial therapy with pulse steroids and a diagnostic delay. Given its pathogenicity among immunocompromised hosts and neurotropism, nocardiosis should be included in the differential diagnosis of acute myelitis among patients with cellular immunodeficiencies. The use of CSF mNGS should be considered to aid in the diagnosis of CNS infections in immunocompromised individuals. Its high cost, restricted accessibility and the potential misinterpretation of contaminants as positive results are important limitations that need to be considered when employing this diagnostic resource.

## Data Availability

The original contributions presented in the study are included in the article/supplementary material, further inquiries can be directed to the corresponding author/s.
